# Effect of a Standardized Extract of *Asparagus officinalis* Stem (ETAS®50) on Cognitive Function, Psychological Symptoms, and Behavior in Patients with Dementia: A Randomized Crossover Trial

**DOI:** 10.1155/2023/9960094

**Published:** 2023-08-22

**Authors:** Takayuki Zenimoto, Mitsuhiko Takahashi

**Affiliations:** Institute of Dementia, Japan Healthcare University, Sapporo 062-0053, Japan

## Abstract

Dementia is a disease of substantial national concern in a superaging society in Japan. Thus, the treatments targeting this disease are of high priority. However, pharmaceutical treatments are under development and invasive. Hence, many alternative treatments, which are less invasive, are tried, and some of them are supposed to work for dementia symptoms. ETAS®50 is one of these treatments. ETAS®50, a standardized extract of the *Asparagus officinalis* stem with heat shock protein-inducing activity, is a functional food. ETAS®50 has antistress, autonomic nerve regulation, and sleep quality improvement effects in humans and could contribute to relieving dementia symptoms. This double-blind crossover pilot trial aimed to examine the effects of ETAS®50. A total of 27 patients with mild-to-moderate dementia between October 2018 and February 2020 were included in the trial. ETAS®50 was consumed for 12 weeks and then a placebo for 12 weeks. A significant difference in the mean score of the severity of symptoms in the Neuropsychiatric Inventory Questionnaire (NPI-Q-a) was observed between the ETAS®50 period (0.56 ± 1.72 points) and placebo period (−0.67 ± 2.34 points) (*p*=0.045). Between-group comparisons with respect to the items of NPI-Q-a also showed a significant decrease in symptoms in the ETAS®50 period compared with the placebo period (*p*=0.015 for agitation and *p*=0.045 for depression). In addition, we observed that scores for apathy tended to improve in the ETAS®50 period (*p*=0.058).

## 1. Introduction

Entry into a superaging society is accelerating worldwide due to the medical system's development and increased life expectancy. The prevalence of dementia among the elderly is increasing along with the number of the elderly. Thus, treatments targeting this disease are of high priority. Efforts to mitigate dementia are being made around the world. The currently available treatments targeting dementia can delay dementia progression and do not serve as direct interventions [1–3]. Furthermore, fundamental treatments targeting amyloid beta-peptides or tau protein and the pharmaceutical prevention of dementia onset remain under development.

Besides pharmacological treatments under development, many nonpharmacological treatments, such as exercise or reminiscence, are attempted and supposed to have some effects. Nonpharmacological treatments are less invasive to the body than pharmacological treatments and are easy to use. Functional foods can also be one of the nonpharmaceutical treatments and have the potential for treatment because of their easy way of intake. They are very beneficial and easy for the elderly with dementia, who have many hazards in their daily lives, such as executive dysfunction and defect of memory.

ETAS®50, a standardized extract of the *Asparagus officinalis* stem, is one of the functional foods. It has been shown to enhance heat shock protein 70 (HSP70)-inducing activity and promote antistress effects in mice [4] and autonomic nerve regulation effects in humans [4, 5]. A human intervention study showed that this treatment improved sleep quality [6–8].

Thus, ETAS®50 is beneficial for stress, autonomic nerve regulation, and sleep quality and is considered effective in treating dementia, which is a form of brain dysfunction [9]. Therefore, we previously conducted a 3-month pilot trial, the prior pilot trial, which is a single-group open-label trial design to verify the clinical efficacy of ETAS®50 for treating mild-to-moderate dementia from November 2016. The results showed that the mean value of the burden scores of the Neuropsychiatric Inventory Questionnaire (NPI-Q-b) ranged from 6.2 ± 5.0 points in the initial point to 3.7 ± 4.1 points in the final point, which showed a significant difference (*p*=0.012). These results suggest that ETAS®50 is effective to a certain extent in improving cognitive function and is even more effective in reducing behavioral and psychological symptoms of dementia (BPSD). No difference was observed between the sex groups (unpublished data).

Therefore, this double-blind crossover pilot trial, the present pilot trial, aimed to further verify these effects and clarify the efficacy of this intervention in ameliorating functionality and symptomology in patients with dementia. To our knowledge, few studies of this type have been conducted on ETAS®50 and these effects are verified.

## 2. Materials and Methods

### 2.1. Methods

This trial was a randomized, double-blind, placebo-controlled, crossover comparison pilot trial. We used five assessment methods, one of which was the Mini-Mental State Examination (MMSE), a 30-point scale for assessing cognitive function [9], which we defined as the primary outcome. The rest of the assessment methods, which we defined as the secondary outcome, included the following: (1) Clock Drawing Test (CDT), a 15-point scale for assessing frontal lobe function [10]; (2) NPI-Q, a 120-point scale for evaluating BPSD, which is designed to be an inventory for caregivers including family members with two categories (NPI-Q-a: severity of symptoms and NPI-Q-b: burden level); (3) Self-Rating Depression Scale (SDS), a depression self-rating scale; and (4) Quality of Life (QOL) questionnaire, a 12-item questionnaire regarding the condition of body and mind made with reference to the Philadelphia Geriatric Center morale scale.

The prior pilot trial did not show any differences between the sex groups. Hence, in the present pilot trial, the participants were divided without regard to sex into two patterns (A and B). The envelope method was adopted as the allocation method. Accordingly, the envelopes were marked with numbers corresponding to the order of the test foods, after which the envelopes were randomly pulled out for each participant who were then allocated into two patterns. This allocation method was administered by the trial staff members.

In the prior pilot trial, NPI-Q-b decreased from 6.2 to 3.7 in patients who received ETAS®50. Assuming similar results, the required sample size for each group would be 32, calculated with a standard deviation of 3.5, a two-sided significance level of 5%, and a power of 80%. A crossover pilot trial with 36 patients would have sufficient power, despite assuming a 10% dropout rate.

Thus, pattern A included 19 participants, and pattern B included 17 participants. Patients took three capsules (100 mg/cps) of ETAS®50 or placebo once a day after lunch for 3 months and were then evaluated for functioning and symptomology at the following time points: initial (before the start of intake), intermediate (after 1.5 months of intake), and final (after 3 months of intake).

The intake of ETAS®50 and placebo was controlled by care workers or families, and the tests were conducted by experienced therapists.

### 2.2. Test Schedule

**Figure 1:**
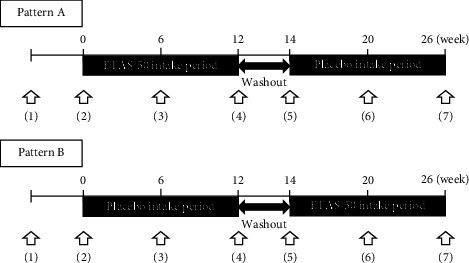
Trial schedule. (1) Explanation; (2), (4), (5), and (7) MMSE, NPI-Q, CDT, SDS, and QOL questionnaire, respectively; (3) and (6) SDS and QOL questionnaire, respectively.

Figure 1 shows the test schedule. In pattern A, the patients received ETAS®50 for 12 weeks, followed by 2 weeks washout period, and then the placebo for 12 weeks. Pattern B was the reverse.

Trial staff members fully explained the trial to the participants before providing their informed consent and taking the test foods. This procedure included explaining how to answer the questionnaire instruments, MMSE, NPI-Q, CDT, SDS, and QOL questionnaire. These implementations were made by the trial staff members. The participants, caregivers, and trial staff members were blinded to the pattern each participant was in during the trial period.

### 2.3. Test Foods

In this pilot trial, ETAS®50 was produced as follows. The asparagus bases were cut using a machine, and their extracts were obtained in a hot water tank. During the process of natural cooling, cellulase and pectinase (plant cell wall degrading enzymes) were added to break down the residue, and the enzymes were inactivated by reheating. The solids were then removed, and after undergoing a sterilization process, the product was made into powder by spray drying.

ETAS®50 and placebo capsules had the same shape and color (dark caramel) to be indistinguishable. ETAS®50 powder was composed of a standardized extract of asparagus extract stem (50 wt% solid content) and dextrin (50 wt%). ETAS®50 powder was manufactured by Amino Up Co., Ltd., and the manufacturing process was conducted in accordance with good manufacturing practice standards for dietary supplements and the ISO9001:2015 and ISO22000:2018 health, safety, and quality control criteria. The placebo powder was composed only of dextrin.

### 2.4. Participants

A total of 36 patients (30 females and 6 males) with mild-to-moderate dementia diagnosed by their physicians, care levels ranging from support required help level 1 to long-term care level 4, and daily life independence levels of I–M were enrolled [11, 12]. Support required help and long-term care are Japanese scales for evaluating the care level for the elderly, based on which they get care. The participants' daily life independence level is also a Japanese scale for evaluating dementia severity. Patients' dementia types included cerebrovascular and Alzheimer's diseases. Users and residents of elderly facilities (i.e., those receiving day services, senior houses, nursing homes, and residences with health and welfare services for the elderly**)** in Sapporo, Japan, were included in this trial.

The exclusion criteria included severe dementia, use of concomitantly banned drugs during the trial period, a history of food allergy, severe liver disease, renal disease, cardiac disease, or hypertension. This trial was conducted between October 2018 and February 2020. The recruitment and follow-up were staggered during the test period.

### 2.5. Statistical Methods

Descriptive statistics were calculated as means and standard deviations for continuous variables and frequency statistics for categorical variables. Between-group comparisons were performed using the Mann–Whitney *U* test, and within-group comparisons were performed using the Wilcoxon signed-rank test. A *p* value <0.05 indicated statistical significance. The *p* value was used to define statistical significance because different tests were used and could, therefore, show significant differences. All statistical tests were conducted using SPSS statistical software (version 24; SPSS, Inc., Chicago, IL, USA).

### 2.6. Ethical Considerations

This pilot trial was conducted in accordance with the Declaration of Helsinki and reviewed and approved by the Research Ethics Committee of Japan Health Care University (approval number 30-13). All participants provided written informed consent before participation.

## 3. Results and Discussion

### 3.1. Results

A total of 36 participants were initially included in the trial. Of the 36 participants, nine were hospitalized or excluded for other reasons as not adverse events (e.g., fractures due to falls or transfer of residence) and had no reference to ETAS®50. Thus, a total of 27 participants (mean age: 87.4 ± 11.6 years) were included in the final analysis. Of the 27 participants, 13 belonged to care level 1, 7 belonged to care level 2 and 7 belonged to other levels. The participants' daily life independence levels were Grade IIb (13 patients), Grade I (7 patients), and Grade IIa (6 patients) (Table 1).

**Figure 2:**
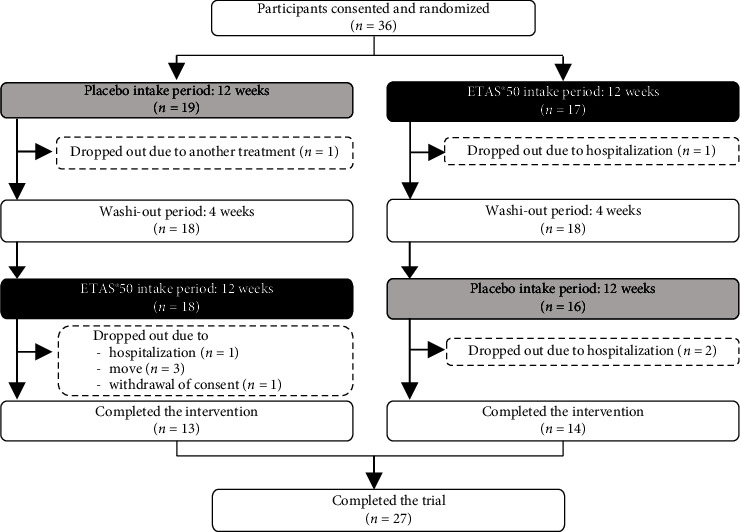
Flow diagram of the trial participants.

Figure 2 shows the flow diagram of the trial participants. The participants were divided into two patterns (pattern A and B). The participants in pattern B took ETAS®50 for 12 weeks and placebo for 12 weeks, with a washout period of 2 weeks between them, and participants of pattern A followed the same procedure in reverse. Meanwhile, nine participants dropped out.

### 3.2. ETAS®50 Improves the Score across Different Assessments

Table 2 shows the score at the initial and final assessment and the difference between the periods (*n* = 27). Regarding the primary outcome, the difference in the MMSE score between the initial point and final point was −0.48 ± 2.33 points (mean ± standard deviation) in the ETAS®50 period and 0.11 ± 2.68 points in the placebo period (*p*=0.420). The MMSE score shows an improvement if the score becomes less.

Regarding the secondary outcome, the difference in the CDT score was −0.28 ± 4.23 points in the ETAS®50 period and −0.1 ± 2.49 points in the placebo period (*p*=0.827). The CDT score shows an improvement if the score becomes more. The mean NPI-Q-a score differed significantly between the ETAS®50 period (0.56 ± 1.72 points) and the placebo period (−0.67 ± 2.34 points) (*p*=0.045). The difference in the NPI-Q-b score was 0.26 ± 1.29 points in the ETAS®50 period and −0.44 ± 2.31 points in the placebo period (*p*=0.472). The NPI-Q score shows an improvement if the score becomes less. The difference in the QOL score was 0.00 ± 3.57 points in the ETAS®50 period and −0.19 ± 4.20 points in the placebo period (*p*=0.903). The QOL score shows an improvement if the score becomes more. The difference in the mean SDS score was 0.37 ± 4.56 points in the ETAS®50 period and 2.07 ± 6.66 points in the placebo period (*p*=0.327).

The mean NPI-Q-a score showed a significant difference (*p*=0.045). However, no significant differences in any other items were observed between the two periods.

### 3.3. Improving for Agitation/Depression in the NPI-Q-a

Table 3 shows the between-group comparisons of NPI-Q-a items because significant differences were observed in NPI-Q-a items. The between-group comparisons of NPI-Q-a items showed significant differences (*p*=0.015 for agitation/aggression and *p*=0.045 for dysphoria/depression). A marginal trend toward apathy was observed (*p*=0.058).

The within-group comparisons of NPI-Q-a items using Wilcoxon's signed-rank test showed a significant difference in agitation in the ETAS®50 period (*p*=0.046). The within-group comparisons do not show the result of the double-blind crossover test but can confirm whether this trial can work or not.

## 4. Discussion

The present pilot trial did not show a significant difference in the MMSE score between the two patterns (i.e., the primary outcome). However, regarding the secondary outcomes, a significant decrease in symptoms of NPI-Q-a was observed in the ETAS®50 period compared with the placebo period (*p*=0.015 for agitation, *p*=0.045 for depression, and *p*=0.058 for apathy). Furthermore, the within-group comparisons between the initial and final scores showed that the symptoms in the ETAS®50 period significantly decreased for agitation (*p*=0.046). A marginal trend toward symptom reduction for apathy (*p*=0.059) and irritability (*p*=0.083) was observed, which suggests that ETAS®50 still exerts some effects against dementia.

The cognitive decline in dementia is said to be due to the accumulation of amyloid-*β* (A*β*) in the brain and neurofibrillary tangles caused by tau protein [13–15]. A*β* forms insoluble A*β* aggregates through the amyloidogenic pathway from its precursor [16]. It has been shown that the C-terminus of the tau protein is cleaved by caspase-3 to facilitate its aggregation [17] and that the production of total A*β* and tau increases during apoptosis [18].

Pharmacotherapies for dementia include rivastigmine, donepezil, and galantamine, which are cholinesterase inhibitors that suppress the breakdown of acetylcholine in the central nervous system, as well as drugs such as memantine, which suppress the release of excess glutamate so that they do not interfere with memory signals [2]. These drugs have the potential to slow down dementia progression or improve memory. However, drugs to stop dementia progression and improve prognosis are still under development. Additionally, antipsychotics such as risperidone, atypical antipsychotic, and anxiolytics may be used when BPSD are severe.

Conversely, nonpharmacological therapies, such as exercise therapy, reminiscence, and music therapy, are also used for treating dementia [3]. Although there is no definitive cure for dementia, many attempts have been made to prevent dementia and slow its progress. These include exercise regimens, dietary interventions, cognitive training, and various alternative and complementary therapies [19]. Functional foods that utilize ingredients from natural agricultural products are also being implemented as interventions, as a potential alternative or augmentation to drugs that use standard chemical ingredients. For example, the flavonol glycosides and terpenoids contained in the *Ginkgo biloba* extract may be effective in improving memory and brain dysfunction by scavenging active oxygen and improving blood circulation [20–22], though this topic remains highly understudied.

ETAS®50, used in this present pilot trial, has previously shown some effects in the brain of different organisms [23]. In animal experiments using mice, it has been confirmed that ETAS®50 intake increases HSP70 in the brain and decreases caspase-3, A*β*, and tau proteins [24]. Furthermore, it has been confirmed that ETAS-fed mice show a reduction in cognitive decline. This effect can be attributed to the fact that Asparaprolin, cyclo(L-leucyl-L-prolyl), cyclo(L-phenylalanyl-L-prolyl), and cyclo (L-tyrosyl-L-prolyl)), in ETAS®50 induces HSP70, which is a factor that suppresses the production of A*β*, the aggregation of tau protein, and the accumulation of caspase-3 related to apoptosis. These effects may avoid A*β* accumulation and tau protein-induced neurofibrillary tangles in the brain, thus suppressing cognitive decline.

Regarding its effects in humans, the prior pilot trial showed some effects in the NPI-Q-b. Similarly, the present pilot trial also showed some effects and tendency in the NPI-Q-a although this was only a secondary outcome. Hence, it is possibly a new finding that consuming ETAS®50 may, at least in part, improve brain function and help combat dementia. Asparagus is a common and familiar vegetable, and if its ingredients are effective in treating dementia, it could contribute substantially to the fight against this disease [25, 26].

These results thus suggest that ETAS®50, a functional food, has a beneficial effect in improving brain function and BPSD and can help attenuate dementia.

In addition, no correlation was observed between the sex of the participants and severity of dementia, perhaps due to the limited number of participants.

## 5. Limitation

Dementia mainly occurs in the elderly who often have other concurrent illnesses, such as high blood pressure, heart failure, and back pain. In this trial, many subjects required treatment and medication for these diseases. Further larger-scale studies are required to evaluate the effects of ETAS®50 on dementia while accounting for the degree of dementia, other diseases, and duration of ETAS®50 intake.

## 6. Conclusions

This pilot trial showed that ETAS®50 possibly has a suppressive effect on agitation and a suppressive tendency for apathy and irritability in dementia. ETAS®50 might help improve BPSD instead of pharmaceutical medicines for people choosing functional foods. This trial showed the potential for treating dementia with minimal physical impact and presented a possible approach for achieving such a treatment.

## Figures and Tables

**Table 1:** Participants' demographic and medical characteristics.

Characteristics	Number	(M, F)
Age (year)		
75–79	4	(1, 3)
80–84	3	(0, 3)
85–89	11	(2, 9)
90–94	6	(0, 6)
95–99	3	(1, 2)
Levels of long-term care or support required		
Support required 1	3	(1, 2)
Support required 2	2	(0, 2)
Care level 1	13	(0, 13)
Care level 2	7	(2, 5)
Care level 3	1	(1, 0)
Care level 4	1	(0, 1)
Care level 5	0	(0, 0)
Daily life independent level		
Grade I	7	(2, 5)
Grade IIa	6	(0, 6)
Grade IIb	13	(2, 11)
Grade IIIa	1	(0, 1)
Grade IIIb	0	(0, 0)
Grade IV	0	(0, 0)
Grade M	0	(0, 0)

Long-term care level (severity): support required 1 < support required 2 < care level 1 < care level 2 < care level 3 < care level 4 < care level 5. Daily life independence level (severity): grade I < grade II (*a* < *b*) < grade III (*a* < *b*) < grade IV < grade M.

**Table 2:** Between-group comparisons of the initial and final scores and differences in scores for each assessment method (*n* = 27).

	ETAS®50 period		
	Initial	Final	Difference
MMSE	21.00 ± 5.19	21.48 ± 5.69	0.48 ± 2.33
CDT	11.60 ± 4.33	11.80 ± 4.51	−0.28 ± 4.23
NPI-Q-a	2.50 ± 3.44	1.96 ± 3.23	−0.56 ± 1.72
NPI-Q-b	2.70 ± 4.64	2.48 ± 4.76	−0.26 ± 1.29
QOL	42.89 ± 5.03	42.89 ± 4.93	0.00 ± 3.57
SDS	34.93 ± 6.35	34.56 ± 7.44	−0.37 ± 4.56

*Placebo period*			*Differencepvalue*
*Initial*	*Final*	*Difference*	

21.11 ± 5.06	21.00 ± 5.97	−0.11 ± 2.68	0.420
11.80 ± 3.75	11.89 ± 4.64	0.09 ± 2.49	0.827
2.20 ± 3.30	2.89 ± 4.85	0.67 ± 2.34	0.045^*∗*^
2.60 ± 4.42	3.00 ± 5.97	0.44 ± 2.31	0.472
43.37 ± 4.72	43.56 ± 4.71	0.19 ± 4.20	0.903
34.89 ± 7.38	32.82 ± 7.02	−2.07 ± 6.70	0.327

^*∗*^ *p* values <0.05 compared to the initial and final scores are considered statistically significant.

**Table 3:** Between-group comparisons of NPI-Q-a items.

NPI-Q-a	ETAS®50 period		
	Initial	Final	Difference
Hallucinations	0.33 ± 0.16	0.26 ± 0.16	−0.07 ± 0.05
Delusions	0.07 ± 0.07	0.07 ± 0.07	0.00 ± 0.00
Agitation/aggression	0.22 ± 0.10	0.15 ± 0.09	−0.07 ± 0.05
Dysphoria/depression	0.26 ± 0.11	0.22 ± 0.10	−0.04 ± 0.04
Anxiety	0.59 ± 0.21	0.33 ± 0.13	−0.26 ± 0.15
Euphoria	0.19 ± 0.11	0.11 ± 0.08	−0.07 ± 0.07
Apathy	0.26 ± 0.13	0.19 ± 0.09	−0.07 ± 0.09
Disinhibition	0.22 ± 0.13	0.22 ± 0.13	0.00 ± 0.00
Irritability	0.22 ± 0.12	0.22 ± 0.12	0.00 ± 0.05
Aberrant motor behavior	0.15 ± 0.10	0.19 ± 0.09	0.04 ± 0.04

*Placebo period*			*Differencepvalue*
*Initial*	*Final*	*Difference*	

0.37 ± 0.15	0.37 ± 0.17	0.00 ± 0.08	0.428
0.04 ± 0.04	0.11 ± 0.11	0.07 ± 0.07	0.317
0.19 ± 0.11	0.33 ± 0.16	0.15 ± 0.07	0.015^*∗*^
0.30 ± 0.12	0.44 ± 0.15	0.15 ± 0.09	0.045^*∗*^
0.56 ± 0.16	0.44 ± 0.15	−0.11 ± 0.10	0.646
0.07 ± 0.05	0.11 ± 0.08	0.04 ± 0.08	0.556
0.19 ± 0.09	0.37 ± 0.12	0.19 ± 0.09	0.058
0.22 ± 0.13	0.26 ± 0.16	0.04 ± 0.06	0.556
0.22 ± 0.12	0.33 ± 0.14	0.11 ± 0.06	0.179
0.07 ± 0.05	0.19 ± 0.11	0.11 ± 0.08	0.542

^*∗*^ *p* values <0.05 compared to the initial and final scores are considered statistically significant.

## Data Availability

The data used in this study are available on reasonable request from the corresponding author.
